# Repetitive Neuromuscular Magnetic Stimulation for Pediatric Headache Disorders: Muscular Effects and Factors Affecting Level of Response

**DOI:** 10.3390/brainsci12070932

**Published:** 2022-07-16

**Authors:** Corinna Börner, Jacob Staisch, Magdalena Lang, Ari Hauser, Iris Hannibal, Kristina Huß, Birgit Klose, Matthias F. Lechner, Nico Sollmann, Florian Heinen, Mirjam N. Landgraf, Michaela V. Bonfert

**Affiliations:** 1 LMU Hospital, Department of Pediatrics—Dr. von Hauner Children’s Hospital, Division of Pediatric Neurology and Developmental Medicine, Ludwig-Maximilians Universität, Lindwurm Str. 4, 80337 Munich, Germany; corinna.boerner@med.lmu.de (C.B.); jacob.staisch@med.uni-muenchen.de (J.S.); magdalena.lang@campus.lmu.de (M.L.); a.hauser@students.uu.nl (A.H.); iris.hannibal@med.uni-muenchen.de (I.H.); kristina.huss@med.uni-muenchen.de (K.H.); birgit.klose@med.uni-muenchen.de (B.K.); matthias.lechner@med.uni-muenchen.de (M.F.L.); florian.heinen@med.lmu.de (F.H.); mirjam.landgraf@med.lmu.de (M.N.L.); 2 LMU Center for Children with Medical Complexity, Dr. von Hauner Children’s Hospital, Ludwig-Maximilians Universität, Lindwurm Str. 4, 80337 Munich, Germany; 3 Department of Diagnostic and Interventional Neuroradiology, School of Medicine, Klinikum Rechts der Isar, Technical University of Munich, Ismaninger Str. 22, 81675 Munich, Germany; nico.sollmann@tum.de; 4 TUM-Neuroimaging Center, Klinikum Rechts der Isar, Technical University of Munich, Ismaninger Str. 22, 81675 Munich, Germany; 5 Department of Diagnostic and Interventional Radiology, University Hospital Ulm, Albert-Einstein-Allee 23, 89081 Ulm, Germany

**Keywords:** migraine, tension-type headache, post-traumatic headache, neuromodulation, neurostimulation

## Abstract

Repetitive neuromuscular magnetic stimulation (rNMS) for pediatric headache disorders is feasible, safe, and alleviates headache symptoms. This study assesses muscular effects and factors affecting response to rNMS. A retrospective chart review included children with headaches receiving six rNMS sessions targeting the upper trapezius muscles. Pressure pain thresholds (PPT) were measured before and after rNMS, and at 3-month follow-up (FU). Mean headache frequency, duration, and intensity within the last 3 months were documented. In 20 patients (14.1 ± 2.7 years), PPT significantly increased from pre- to post-treatment (*p* < 0.001) sustaining until FU. PPT changes significantly differed between primary headache and post-traumatic headache (PTH) (*p* = 0.019–0.026). Change in headache frequency was significantly higher in patients with than without neck pain (*p* = 0.032). A total of 60% of patients with neck pain responded to rNMS (≥25%), while 20% of patients without neck pain responded (*p* = 0.048). 60% of patients receiving rNMS twice a week were responders, while 33% of patients receiving rNMS less or more frequently responded to treatment, respectively. Alleviation of muscular hyperalgesia was demonstrated sustaining for 3 months, which was emphasized in PTH. The rNMS sessions may positively modulate headache symptoms regardless of headache diagnosis. Patients with neck pain profit explicitly well. Two rNMS sessions per week led to the highest reduction in headache frequency.

## 1. Introduction

Primary headache disorders like migraine and tension-type headache (TTH) are highly prevalent in kids and adolescents, and they represented the second most disabling conditions in 10- to 24-year-old individuals in 2019 [1,2,3,4]. However, these conditions are considerably underdiagnosed [5,6,7,8,9]. Another common entity is post-traumatic headache (PTH) with the heterogeneous character of migraine-like, TTH-like, daily, or continuous headaches [10,11,12,13,14].

In the pathophysiology of these headache disorders, the trigemino-cervical complex (TCC) plays an important role [15,16]. Upper cervical afferents in the neck muscles transmit nociceptive and proprioceptive information to the caudal trigeminal nucleus. There, information is converged with sensory information from trigeminal branches in the head/face area and delivered to pain processing centers in the brain [15,17,18]. Consequently, the TCC represents the key framework of the interplay of peripheral and central mechanisms of pain perception, processing, perpetuation, and sensitization [15,18,19,20].

Within this concept, muscular involvement accompanying headache disorders comprises reports of neck pain or tension as well as findings during manual palpation of the short neck and upper trapezius muscles (UTM) [21,22,23,24]: next to muscular imbalance, restricted range of motion, or hyperalgesia, involvement of these muscles encompasses the presence of myofascial trigger points (mTrP), defined by taut bands, hypersensitive spots, and referred sensation during manual palpation [15,21,22,23,24,25,26,27,28,29,30,31,32,33,34,35]. In addition, PTH is frequently associated with muscular symptoms due to a whiplash-like component by rotational mechanical forces and a subsequent dysregulation of muscle tone in the neck muscles, which can present with neck pain and similar muscular signs as described for migraine and TTH [12,36,37,38].

A multimodal therapeutic strategy approaching the burdensome migraine, TTH, and PTH in children, calls for non-invasive, non-pharmacological, safe, and well-accepted treatments [39]. Neurostimulation of the cranial nerves represents one of the currently increasingly investigated approaches in adult neurology [40]. As muscular involvement is frequently diagnosed in pediatric headache disorders, a personalized treatment protocol applying repetitive neuromuscular magnetic stimulation (rNMS) targeting the UTM has been recently developed, demonstrating a feasible and safe set-up process and the effective alleviation of headache symptoms [41]. It is hypothesized that the clinical effects of rNMS in headache patients are achieved through a modulation of central pain processing networks. By electromagnetic stimulation above the UTM, sensory input via the upper cervical afferents (C1-C3) transferring to the TCC is increased by direct and indirect stimulation [42,43,44,45].

The aims of this retrospective analysis were (1) to assess the local effects on the (peripheral) muscular level of this rNMS treatment, and (2) to investigate whether the specific headache disorder (primary headache or PTH), the presence of neck pain, and the time frame rNMS is delivered in, may affect the response with regard to muscular and headache symptoms.

## 2. Materials and Methods

### 2.1. Ethics

The study was approved by the institutional review board of the medical faculty of the University of Munich (LMU; vote 21-0574).

### 2.2. Study Design

During chart review, 23 patients were identified, who had a diagnosis of (1) episodic migraine, (2) episodic TTH, (3) mixed-type headache [46], or (4) PTH [14,47] and received rNMS treatment in our outpatient pediatric headache clinic between August 2020 and May 2021. A description of the study design is detailed in Staisch et al. (2022) [41].

### 2.3. rNMS Intervention

All patients received rNMS delivered by an eMFieldPro system (Zimmer MedizinSysteme GmbH, Neu-Ulm, Germany; CE Nr 0123). Stimulation was targeted to the UTM bilaterally in 6 consecutive sessions during two to three weeks. Each side was stimulated for 15 min consisting of 7420 pulses (20 Hz, 7 s ON time, 10 s OFF time) with a duration of 250 μs by a coil creating a magnetic field of 2.5 T maximum (7.6 cm diameter of the copper winding). The starting side was alternated in each session and the stimulation intensity was individually set to a comfortable level. Detailed descriptions of the rNMS setup and stimulation protocol have been recently published [41].

### 2.4. PPT Assessments

Before and after each rNMS session, mechanical algometry was performed using a hand-held analogous algometer (Wagner instrument, Greenwich, CT, USA) to determine the patient’s pressure pain thresholds (PPT) above the UTM. The PPT is defined as the cut-off weight between the perception of pressure and the perception of pressure-induced pain [31,48]. Patients were seated on a roller stool with their hands resting on their laps. Reference points were marked at 1/3 and 2/3 of the distance from C7 to the acromion above the left and right UTM. The algometer was placed perpendicular on the skin with steadily increasing pressure until the patient indicated that the PPT was reached. Measurements were repeated three times in total, always starting with the lateral point on the right, continuing with the left lateral point, and resuming in the same order with the medial points. This protocol ensured that sufficient time had passed between the three measurements on each reference point [48]. The measurement was performed again during 3-months follow-up (FU) evaluation.

### 2.5. Headache Characteristics

Headache frequency, headache intensity (minimum and maximum), and headache duration regarding the headaches in the last 3 months were documented before treatment and at FU. Patients were classified according to responder rates (≥25%, ≥50%, ≥75%) based on their relative reduction in headache frequency. While headache changes in the total sample have been previously analyzed [41], this manuscript examines differences in headache changes among subgroups: (1) headache diagnosis, (2) neck pain, and (3) treatment time frame.

### 2.6. Data Management

Paper-based clinical report forms and customized questionnaires were used to document rNMS interventions and FU examinations. Data were anonymized, entered into Microsoft Excel data sheets (Microsoft Office Professional Plus 2016, Microsoft, Redmond, WA, USA), and plausibility of data was checked by at least two independent analysts. The algometer’s maximum pressure was 10 kg/cm^2^. If no pain was indicated when that pressure was reached, 10 kg/cm^2^ was defined as the PPT [49]. Headache frequency was assessed as headache days per month, headache duration as hours, and headache intensities as 10-point visual analogue scale (VAS).

### 2.7. Statistical Analysis

Microsoft Excel (Microsoft Office Professional Plus 2016, Microsoft, Redmond, WA, USA) and SPSS (version 25/26; IBM SPSS Statistics for Windows, Armonk, NY, USA) were used for statistics. The level of statistical significance was set at α = 0.05. Shapiro–Wilk tests checked for normality of the headache variables and PPT measurements. The Pearson correlation between the time from treatment to FU examinations and the PPT at FU examinations above each reference point were calculated. Since no correlations were observed (left lateral: r = −0.057, left medial: r = 0.084, right medial: r = 0.008, right lateral: r = 0.052), time from treatment to FU examinations was not considered as a covariate for the following analyses.

Differences in the PPT between pre-, post-, and FU assessments in the total sample were assessed with a repeated-measures analysis of variance (ANOVA). The homogeneity of variances at different time points was assessed by Mauchly’s test and was given for all analyses. Bonferroni corrections were used for adjustment for multiple comparisons. Effect sizes were calculated using eta squared.

In addition, the following subgroup analyses were performed: (1) primary headache group vs. PTH group, (2) neck pain group vs. no neck pain group, and (3) different treatment time frame groups (<2x/week, 2x/week, >2x/week). Comparisons of the PPT above each reference point between the groups were calculated with two-way repeated measures ANOVA with time as within-factor and group as between-factor. Bonferroni (within) and Tukey (between) corrections were used for adjustment for multiple comparisons. Differences in the relative change of headache characteristics and the PPT from baseline to FU examinations between the headache diagnosis groups as well as the neck pain groups were assessed using two-samples *t*-tests and Mann–Whitney U tests. Differences in the relative change in headache characteristics and PPT from baseline to FU examinations between the treatment time frame groups were assessed using one-way ANOVA. Pearson correlations between the time since headache onset or trauma and the relative change in headache frequency were calculated for the headache diagnosis, neck pain, and treatment time frame comparisons. Sample sizes for the comparisons are listed in Table 1.

## 3. Results

### 3.1. Subjects

A total of 23 patients completed the rNMS intervention, of whom 5 patients received a second block of rNMS on average 104.2 ± 32.8 days (range: 73–167 days) after the first intervention, resulting in 28 rNMS interventions in total. Two patients were lost to FU and one patient was identified as an outlier based on a late FU as the FU time (210 days after intervention) lied more than 3 standard deviations above the mean FU time of the sample (91.7 ± 26.7 days). Therefore, data from 20 patients receiving 25 rNMS interventions were analyzed. Since three patients received FU examination via telephone calls, data from 17 patients receiving 22 rNMS interventions were included in the PPT analysis. Details on the sample sizes for analysis are given in Table 1.

### 3.2. Patient and Treatment Characteristics

Patients were on average 14.1 ± 2.7 years old, and 12 patients were females (60%). Diagnoses included migraine without aura and TTH (n = 8, 40%), migraine with aura (n = 2, 10%), migraine without aura (n = 2, 10%), migraine with aura and TTH (n = 1, 5%), and PTH (n = 7, 35%). Acute medication was used by patients in 12 cases (48%) and a pharmacoprophylaxis with magnesium in 11 (44%). In the 3 months before the intervention, physiotherapy was obtained in 12 cases (48%), and was continued during the intervention in 5 (20%). During the 3 months after the intervention, in 4 cases (16%) physiotherapy was continued, and in 5 cases (20%) started. Neck pain as a general complaint besides headaches was indicated 15 times (60%) at the beginning of a rNMS treatment block. For the left UTM, mean stimulation intensity was 25.0% ± 11.6 of the maximum stimulator output and for the right UTM 25.8% ± 11.3 of stimulator output. A detailed description of the study population can be found in Staisch et al. (2022) [41].

### 3.3. Pressure Pain Thresholds

**Figure 1:**
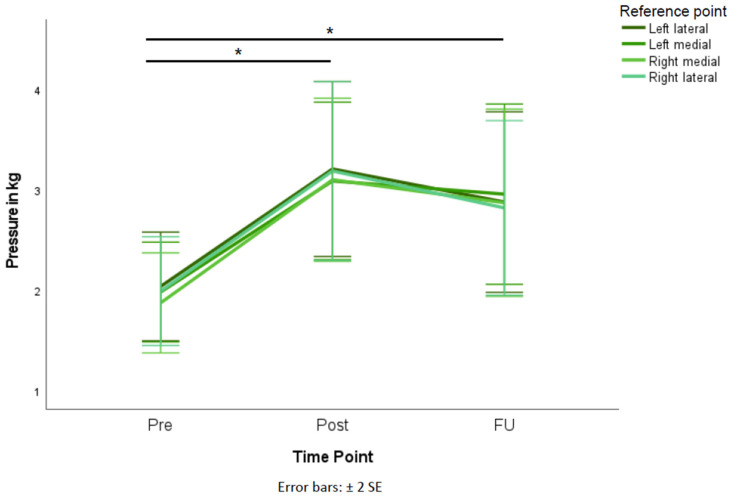
PPT results above each reference point before the first rNMS session (pre), before the last rNMS session (post), and at FU (n = 22 with complete FU). Significant differences are marked with an asterisk (*) and apply to changes at all 4 reference points. Error bars are depicted as ±2 standard errors (SE).

**Figure 2:**
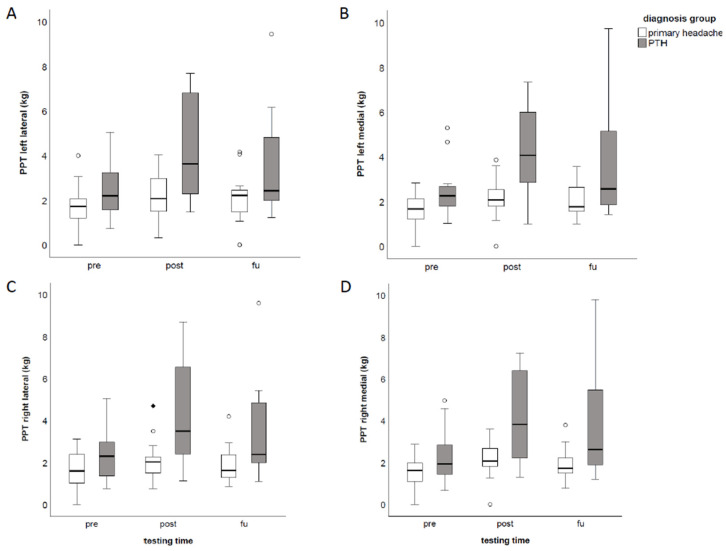
Comparison of PPT changes across time between the primary headache group (white) and PTH group (grey). (**A**) Comparison of PPT above left lateral reference point, (**B**) Comparison of PPT above left medial reference point, (**C**) Comparison of PPT above right lateral reference point, (**D**) Comparison of PPT above right medial reference point. Abbreviations: PPT: pressure pain threshold, PTH: post-traumatic headache, pre: before the first rNMS session, post: before the last rNMS session, FU: follow-up.

Comparisons of the PPT above each reference point resulted in significant differences for all reference points over time (left lateral: *p* < 0.001, η^2^ = 0.318; left medial: *p* < 0.001, η^2^ = 0.351; right medial: *p* < 0.001, η^2^ = 0.363; right lateral: *p* < 0.001, η^2^ = 0.311). PPT were increasing from pre- to post-treatment assessments and did not significantly change from post-treatment to FU examinations (Table 2, Figure 1). PPT changes above each reference point over time significantly differed between the primary headache group and the PTH group (left lateral: *p* = 0.026, η^2^ = 0.225; left medial: *p* = 0.019, η^2^ = 0.247; right medial: *p* = 0.019, η^2^ = 0.244; right lateral: *p* = 0.020, η^2^ = 0.241; Table 3, Figure 2). When comparing patients with and without neck pain, no significant differences in PPT changes across time were observed between the groups (left lateral: *p* = 0.688; left medial: *p* = 0.807; right medial: *p* = 0.765; right lateral: *p* = 0.520). Regarding the comparison of different treatment time frames, PPT changes over time did not significantly differ between the three groups (left lateral: *p* = 0.146; left medial: *p* = 0.262; right medial: *p* = 0.187; right lateral: *p* = 0.282).

### 3.4. Headache Characteristics

**Figure 3:**
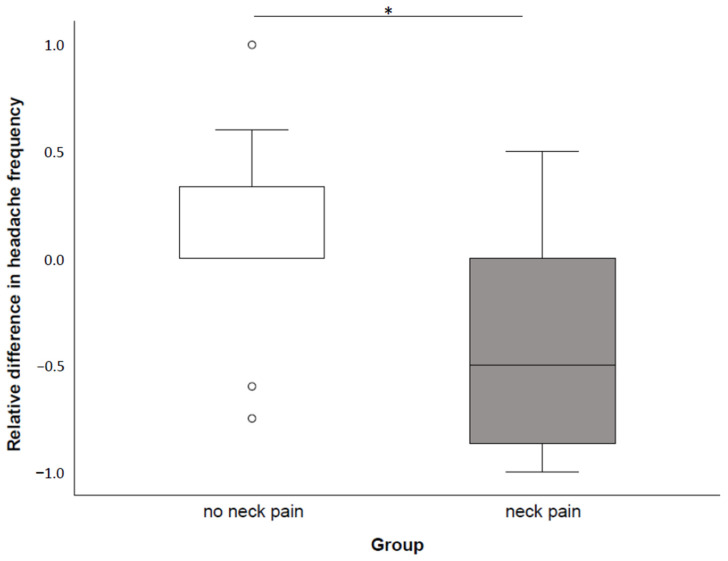
Relative difference in headache frequency (days/month) from baseline to follow up after rNMS intervention in patients with and without neck pain. Significant differences at α = 0.05 are marked with an asterisk (*).

When comparing patients with and without neck pain at baseline, the change in headache frequency was significantly higher in the neck pain group (t = 2.29, *p* = 0.032, confidence interval 0.04–0.89; Figure 3). Changes in headache intensity (minimum: *p* = 0.434; maximum: *p* = 0.434) and duration (*p* = 0.511) did not significantly differ between these groups. No significant differences were observed for the changes in headache characteristics in the headache diagnosis (headache frequency: *p* = 0.191; minimum headache intensity: *p* = 0.679; maximum headache intensity: *p* = 0.770; headache duration: *p* = 0.923) and treatment time frame comparisons (headache frequency: *p* = 0.462; minimum headache intensity: *p* = 0.600; maximum headache intensity: *p* = 0.059; headache duration: *p* = 0.318).

Regarding the responder rate classification (≥25%, ≥50%, ≥75%), 43% of patients with primary headaches were responders (≥25%) with 14% being classified as 75% responders (Table 4). In the PTH group, 46% of patients were responders and all were categorized as 75% responders. Furthermore, 60% of patients with neck pain responded to rNMS (≥25%), while 20% of patients without neck pain were responders (≥25%, *p* = 0.048). In addition, 60% of patients receiving rNMS twice a week were responders (≥25%), while 33% of patients receiving rNMS less or more than twice a week responded to treatment (≥25%), respectively.

No statistically significant correlation for time since headache onset / time since trauma and the relative mean change in headache frequency was found in the primary headache group (r = −0.05, *p* = 0.857), nor in headache frequency in the PTH group (r = 0.23; *p* = 0.492; Table 5).

## 4. Discussion

This retrospective analysis evaluated the muscular effects of rNMS targeting the UTM in children and adolescents with headache disorders. As an additional goal, we investigated whether the type of headache disorder, the presence of neck pain, and the time frame of rNMS affected the change of muscular and headache symptoms.

With regard to muscular effects, muscular hypersensitivity decreased from pre- to post-treatment assessment and was sustained until FU examinations on a lower level than at baseline. No significant differences in PPT changes were found between neck pain and treatment time frame groups, respectively. Thus, the response of the PPT seems not to depend on the presence of neck pain, nor on the time frame rNMS is applicated in. When compared to healthy controls, migraine patients demonstrated a pronounced pressure pain sensitivity translating to a lower PPT in the neck and shoulder region in previous studies [50,51,52]. Our analysis suggests a higher level of muscular hypersensitivity in pediatric headache patients before the rNMS intervention with a similar or even lower PPT compared to adults with migraine [51]. After rNMS intervention, PPT were comparable to or even higher than the PPT of healthy adults [51]. This finding may underline that with the application of rNMS to UTM, muscular hypersensitivity in headache patients can reach a “healthy” level. As decreased PPT are interpreted as a sign of sensitization of the trigemino-cervical nucleus caudalis [53], rNMS is likely to exert a desensitization effect on the TCC. Our findings regarding PPT are in accordance with previous studies that explored the muscular effects of rNMS in young adults with frequent episodic migraine receiving six rNMS sessions (stimulation protocol: 15 min/side, 20-Hz frequency, 15 s ON time, and 30 s OFF time) [48,54,55]. While a significant increase in the pre- and post-treatment algometry-based PPT was reported over the course of the intervention in the pilot study [55], an increase in PPT was bilaterally observed but only reached statistical significance in the left UTM in a later study [48]. However, PPT were only measured locally, directly above an active mTrP, and no reference points were explored in these previous studies [48,55].

Moreover, the current study investigated the possible impact of headache diagnosis, presence of neck pain, or the treatment time frame on the clinical efficacy of rNMS. No significant differences regarding changes in headache symptoms or responder rates were observed between patients with primary headaches and PTH, thus indicating a similar response to rNMS irrespective of the distinct headache diagnosis. However, it should be noted that all PTH patients showed at least 75% reduction in headache frequency, which is an important clinical finding on the individual patient’s level. Patients with neck pain as a general complaint in addition to their headaches demonstrated a greater reduction in headache frequency than patients without neck pain, with significantly higher responder rates. Following the concept of the TCC, rNMS is expected to have a greater impact on headache characteristics, like headache frequency, in patients with neck pain [31,44]. No significant differences regarding changes in headache symptoms were present between groups of different treatment time frames. However, the highest responder rates were documented in patients receiving rNMS twice per week (60% responders), which is why a treatment protocol with two sessions per week might be preferred.

Neurostimulation by rNMS represents a novel approach to acute and preventive treatment in primary headache disorders. Transcutaneous supraorbital nerve stimulation (tSNS) [56,57,58], transcutaneous occipital nerve stimulation (tONS) [59,60], and transcutaneous vagus nerve stimulation (tVNS) [61,62] have previously been investigated within this context [40]. Regarding PTH, no adequate treatments have yet been established, which is why treatment of PTH is commonly based on research in primary headache disorders [38,63].

In comparison to the above-mentioned techniques that stimulate cranial nerves directly, rNMS is not only safe but also well accepted among patients, which is an important factor especially in the pediatric field [40,41]. The key benefit of rNMS is the muscular approach via the UTM, which is clinically involved in the pathophysiology of headache disorders via the TCC [15,16]. Therefore, the choice of the UTM as a stimulation target can be easily explained to the patients (especially in cases of reported neck pain or muscular symptoms together with headache symptoms), who themselves directly experience the stimulation at the local level and control the treatment by being able to adjust stimulation intensity. Hence, rNMS represents a personalized neurostimulation approach from the bottom-up by simultaneously modulating muscular and central pain processing (network reorganization) [40]. However, studies evaluating the efficacy of rNMS differ in methodology so far, making the use of guidelines for quality improvement of rNMS methods, and thus better comparability of studies, inevitable in the future [64].

Some limitations apply to the current study. First, it reports data collected during everyday routines within our clinical setting. Patients could have received other therapies like physiotherapy or psychological interventions in parallel to the rNMS intervention within the multimodal treatment regimen. As the setting/placebo effects might be increased in the pediatric field and for medical device treatments, this may have influenced the effects as well [65,66]. Thus, future studies with a prospective and controlled design investigating larger patient samples are needed. Second, the period of analyzed rNMS interventions was during the COVID-19 pandemic. Therefore, the life of children and adolescents changed drastically as, among other things, schools were closed from time to time, digital learning time during home schooling strongly increased, and contact with peers and friends were not possible. The possible impact of COVID-19-related lifestyle changes on headaches in our sample cannot be estimated, as lifestyle factors are known to influence headache symptoms [67]. Specifically, for an Italian cohort it has been reported that school closure was related to a reduction in headache frequency and intensity in school children with headaches [68]. Such effects should be taken into consideration by future studies in a controlled setting.

## 5. Conclusions

Reduced muscular hyperalgesia after rNMS was demonstrated in pediatric patients with headache disorders, which was sustained up to weeks to months. This effect was particularly emphasized in patients with PTH. In addition, rNMS seems to positively modulate headache symptoms regardless of the specific headache diagnosis. Patients with neck pain profit explicitly well from the intervention. Regarding treatment time frame, two rNMS sessions per week led to the highest reduction in headache frequency. Given the framework of the trigemino-cervical complex, rNMS targeting the UTM most likely acts via neuromodulation of nociceptive processing at the central level.

## Figures and Tables

**Table 1:** Sample sizes for PPT analysis and subgroup analyses.

Group	n (Patients)	n (Interventions)
Total sample	20	25
PPT analysis (total sample)	17	22
Headache diagnosis analysis		
Primary headaches	13	14
PTH	7	11
Neck pain analysis		
With neck pain	13	15
Without neck pain	7	10
Treatment time frame analysis		
<2x/week	8	9
2x/week	7	10
>2x/week	5	6

Abbreviations: PPT pressure pain threshold, PTH post-traumatic headache.

**Table 2:** Comparison of PPT above each reference point before the first rNMS session (pre), before the last rNMS session (post), and at FU.

PPT	Test Values			Mean (SD)			Post-Hoc Test
	F	*p*	η^2^	Pre	Post	FU	*p*
Left lateral	9.77	<0.001 *	0.318	2.00 (1.37)	3.28 (2.21)	2.87 (2.11)	
Pre-post							0.001 *
Pre-FU							0.034 *
Post-FU							0.415
Left medial	11.38	<0.001 *	0.351	1.96 (1.27)	3.17 (1.99)	2.95 (2.11)	
Pre-post							0.002 *
Pre-FU							0.007 *
Post-FU							0.988
Right medial	11.98	<0.001 *	0.363	1.83 (1.26)	3.17 (2.06)	2.95 (2.11)	
Pre-post							0.001 *
Pre-FU							0.004 *
Post-FU							0.788
Right lateral	9.49	<0.001 *	0.311	1.94 (1.37)	3.24 (2.25)	2.81 (2.04)	
Pre-post							0.002 *
Pre-FU							0.020 *
Post-FU							0.510

Differences in PPT above each reference point were analyzed using repeated-measures ANOVAs and Bonferroni post-hoc comparisons. Significant differences at α = 0.05 are marked with an asterisk (*). Abbreviations: PPT: pressure pain threshold (in kg), pre: before the first rNMS session, post: before the last rNMS session, FU: follow-up, rNMS: repetitive neuromuscular magnetic stimulation, SD: standard deviation, F: ANOVA test statistic, η^2^: effect size eta-squared.

**Table 3:** Comparison of PPT changes across time between the primary headache group and PTH group.

PPT	Test Values			Mean (SE)		
	F	*p*	η^2^	Pre	Post	FU
Left lateral						
Headache diagnosis	5.82	0.026 *	0.225			
Time	10.50	<0.001 *	0.344			
Time * headache diagnosis	2.57	0.089	0.114			
Primary headache				1.49 (0.39)	2.13 (0.58)	2.07 (0.60)
PTH				2.50 (0.39)	4.43 (0.58)	3.68 (0.60)
Left medial						
Headache diagnosis	6.56	0.019 *	0.247			
Time	11.62	<0.001 *	0.368			
Time * headache diagnosis	1.45	0.247	0.068			
Primary headache				1.38 (0.35)	2.13 (0.52)	2.13 (0.60)
PTH				2.54 (0.35)	4.20 (0.52)	3.78 (0.60)
Right medial						
Headache diagnosis	6.47	0.019 *	0.244			
Time	12.66	<0.001 *	0.388			
Time * headache diagnosis	2.19	0.125	0.099			
Primary headache				1.33 (0.36)	2.11 (0.54)	1.93 (0.61)
PTH				2.34 (0.36)	4.22 (0.54)	3.80 (0.61)
Right lateral						
Headache diagnosis	6.34	0.020 *	0.241			
Time	9.94	<0.001 *	0.332			
Time * headache diagnosis	2.00	0.148	0.091			
Primary headache				1.41 (0.39)	2.12 (0.60)	1.97 (0.57)
PTH				2.46 (0.39)	4.37 (0.60)	3.66 (0.57)

Differences in PPT changes above each reference point between groups were analyzed using two-way repeated measures ANOVAs with time as within-factor (pre, post, FU) and group as between-factor (primary headache, PTH). Multiple comparison correction was done using the Bonferroni (within) and Tukey (between) procedures. Significant differences at α = 0.05 are marked with an asterisk (*). Abbreviations: PPT: pressure pain threshold (in kg), PTH: post-traumatic headache, pre: before the first rNMS session, post: before the last rNMS session, FU: follow-up, SD: standard deviation, F: ANOVA test statistic, η^2^: effect size eta-squared.

**Table 4:** Classification in responder rates based on the relative headache frequency reduction from pre-assessment to FU for the headache diagnosis, neck pain, and treatment time frame group comparisons.

Responder Rate	Headache Diagnosis Comparison n (%)		Neck Pain Comparison n (%)		Treatment Time Frame Comparison n (%)		
	Primary Headache	PTH	Neck Pain	No Neck Pain	<2x/Week	2x/Week	>2x/Week
75% responder	2 (14.3)	5 (45.6)	6 (40)	1 (10)	2 (22.2)	4 (40)	1 (16.7)
50% responder	5 (35.7)	5 (45.6)	8 (53.3)	2 (20)	2 (22.2)	6 (60)	2 (33.3)
25% responder	6 (42.9)	5 (45.6)	9 (60) *	2 (20) *	3 (33.3)	6 (60)	2 (33.3)
No responder	8 (57)	6 (54.6)	6 (40) *	8 (80) *	6 (66.7)	4 (40)	4 (66.7)

Differences in responder rates between groups were assessed using Chi-square tests. Significant differences at α = 0.05 are marked with an asterisk (*).

**Table 5:** Pearson’s correlations between the time since headache onset or trauma and the relative mean change in headache frequency per headache diagnosis, neck pain, and rNMS time frame groups.

Group	Correlation r	*p*
Headache diagnosis groups		
Primary headache ^a^	−0.05	0.857
PTH ^b^	0.23	0.492
Neck pain groups		
Neck pain ^a^	−0.04	0.881
No neck pain ^a^	0.34	0.334
Treatment time frame groups		
<2x/week ^a^	0.53	0.139
2x/week ^a^	−0.03	0.943
>2x/week ^a^	0.03	0.955

^a^ Correlation using time since headache onset, ^b^ correlation using time since trauma.

## Data Availability

The data presented in this study are available on request from the corresponding author. The data are not publicly available due to the sensitive character of pediatric clinical data.
